# Development of an Ambulatory Biofeedback App to Enhance Emotional Awareness in Patients with Borderline Personality Disorder: Multicycle Usability Testing Study

**DOI:** 10.2196/13479

**Published:** 2019-10-15

**Authors:** Youri PMJ Derks, Randy Klaassen, Gerben J Westerhof, Ernst T Bohlmeijer, Matthijs L Noordzij

**Affiliations:** 1 Department of Psychology, Health, and Technology University of Twente Enschede Netherlands; 2 Scelta, Center for Treatment of Personality Disorders GGNet, Mental Health Institute Apeldoorn Netherlands; 3 Department of Human Media Interaction University of Twente Enschede Netherlands

**Keywords:** mental health, borderline personality disorder, mobile health, emotional awareness, ambulatory biofeedback, design science, user centered design, iterative prototype testing

## Abstract

**Background:**

Patients with borderline personality disorder experience great difficulties in regulating their emotions. They often are unable to effectively detect their emotional arousal and struggle to timely apply learned techniques for emotion regulation. Although the use of continuous wearable biofeedback has been repeatedly suggested as an option to improve patients’ emotional awareness, this type of app is not yet available for clinical use. Therefore, we developed an ambulatory biofeedback app named Sense-IT that can be integrated in mental health care.

**Objective:**

The aim of the study was to develop an ambulatory biofeedback app for mental health care that helps with learning to better recognize changes in personal emotional arousal and increases emotional awareness.

**Methods:**

Using several methods in a tailored User Centred Design (UCD) framework, we tested the app’s usability and user experience (UX) via a cyclic developmental process with multiple user groups (patients, therapists, and UCD experts; 3-5 per group, per cycle).

**Results:**

The process resulted in a stable prototype of the app that meets most of the identified user requirements. The app was valued as useful and usable by involved patients, therapists, and UCD experts. On the Subjective Usability Scale (SUS), the patients rated the app as “Good” (average score of 78.8), whereas the therapists rated the app as “OK” (average score of 59.4). The UCD experts judged the app’s overall usability as between “OK” and “acceptable” (average score of 0.87 on a cognitive walkthrough). As most critical usability problems were identified and addressed in the first cycle of the prototyping process, subsequent cycles were mainly about implementing new or extending existing functions, and other adjustments to improve UX.

**Conclusions:**

mHealth development within a clinical mental health setting is challenging, yet feasible and welcomed by targeted users. This paper shows how new mHealth interventions for mental health care can be met with enthusiasm and openness by user groups that are known to be reluctant to embrace technological innovations. The use of the UCD framework, involving multiple user groups, proved to be of added value during design and realization as evidenced by the complementary requirements and perspectives. Future directions on studying clinical effectiveness of the app, appliance of the app in other fields, and the implications of integration of the app for daily practice in mental health are discussed.

## Introduction

### Context

Borderline personality disorder (BPD) is a psychological disorder that influences all domains of life. It is characterized by a pervasive pattern of unstable relations, a distorted self-image, and profound difficulties in regulation of one’s emotions [1]. Self-harming behaviors are common [2-5]. A lack of emotional awareness or the ability to timely recognize the onset of emotions and their increasing or decreasing intensity plays a role in emotional instability and dysregulated and self-harming behaviors in BPD [6-11]. Patients with BPD seem to especially have less focus on the level of emotional arousal than controls [12].

Perception of internal bodily states is found to be of significant importance to subjective experience, awareness, labeling, and understanding of emotional processes [13-16]. Interestingly, people with low emotional awareness, in general, *do* respond to emotional triggers physiologically, and to a certain extent, behaviorally, but lack the experience of the emotion—the *feeling* [14,17,18]. Therefore, available evidence suggests that treatment of low emotional awareness in BPD should focus on bodily signals and could very well involve biofeedback [6,12-23]. Although there are indications that emotional awareness can improve with psychological interventions [24], development and testing these interventions is in its infancy [25,26]. From literature and information gathered from patients and professionals, we identified a need to improve the treatment of low emotional awareness in BPD [19,24,27].

We started a project to develop a biosensor-informed e-coaching app on emotional awareness in the challenging environment of a psychiatric ward for patients with severe BPD. We gave the app the name *Sense-IT*, as it refers to both its intended purpose for patients (*sense it*) and its *technological nature* (IT: information technology). Importantly, it should help its users to learn to better recognize changes in their physiological and, with that, emotional arousal and thus increase emotional awareness. To the best of our knowledge, this project is one of the first in providing ambulatory biofeedback to this group of users. We previously developed an initial prototype [28]. The aim of this study was to complete the next step and deliver a working version of the Sense-IT app that is deemed useful and usable by 3 important groups of stakeholders: patients with BPD and low emotional awareness, mental health professionals working with these patients, and experts on user-centered design (UCD).

### Initial Prototype

We decided to design our app for wearable technology that comes equipped with essential biosensor technology that is widely available for consumers, is affordable, and runs on a mature operating system (OS) that offers easy-to-use app programming interfaces (APIs) by which one can develop native apps and access sensors directly. After a small preliminary study with main stakeholders and users on acceptable, usable, and nonstigmatizing hardware (unpublished), we decided to use a smartwatch and mobile phone. Next, we decided on developing an app for Google’s Android (including Wear) OS. As a mature, easy-to-access OS, it has a broad base of support by manufacturers, software developers, and users alike. Most smartwatches (at the time) came equipped with a photoplethysmogram (PPG) sensor and accelerometer. The PPG sensor is used for measuring heart rate (HR). After studying the literature on the use of biosignals for affect detection, we built an algorithm that takes the HR data of the user to calculate a *physiological correlate of emotional arousal* (*PCEA*). HR, similar to HR variability (HRV) and electrodermal activity (EDA), is triggered by the sympathetic nervous system [29,30]. Typical consumer wrist-based HR monitors provide a fairly accurate measurement of HR even when deployed during physical activity and movement [31].

**Figure 1:**
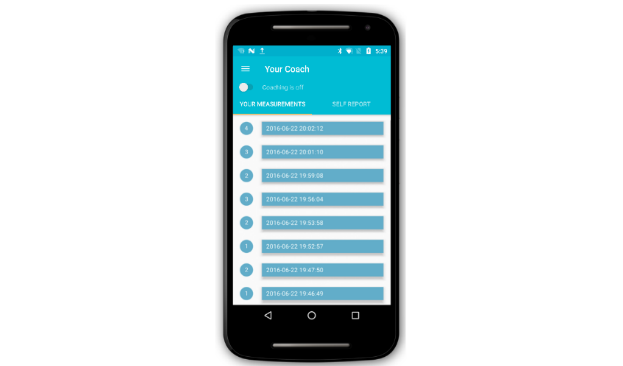
Sense-IT mobile phone graphical user interface, first version. The yellow switch starts and stops the measuring of heart rate (HR) via the smartwatch. Each recorded change in level of HR (or physiological correlate of emotional arousal [PCEA]) is represented a blue bar in the white area of the screen. The date and time of the change are displayed within the blue bar, and the level of the PCEA appears as a number in the circle left of the bar.

**Figure 2:**
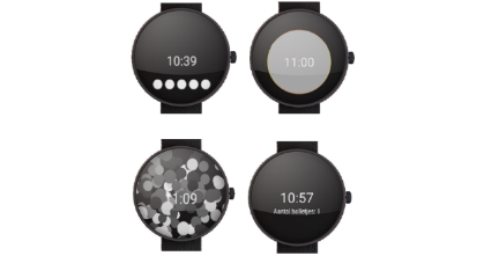
Sense-IT watch faces, first version. The watch faces each expressed changing levels of HR/PCEA by changing either the circular elements (ie, more circles or a bigger circle), or simply indicating a numerical level (from 1 – 10).

The first version of the app was built by one of the authors (RK) and 2 graduate students in Human Media Interaction. The outcomes of previous design cycles served as the main sources of input for programming (refer to the study by Derks et al [28]). Visual design was kept low in complexity so that the app’s graphical user interface (GUI) is easy to interpret by the user yet remains nondescript to the environment (see Figure 1). To cater to the expressed need for discreet and unobtrusive coaching, audio signals were deliberately not implemented. Instead, the app was programmed to give tactile feedback via the vibration motor in the smartwatch. Users have direct access to a proxy of the current level of the PCEA via continuous HR measurements on both the smartwatch and mobile phone. On the smartwatch, users have the option to choose from 4 different watch faces. The mobile phone app provides an overview of the recent measurements. At higher levels of arousal (stages 4 and 5 out of 5), the user receives a textual prompt, intended to stimulate deliberate reflection on one’s current status of emotional arousal. See Figure 2 for an impression.

**Figure 3:**
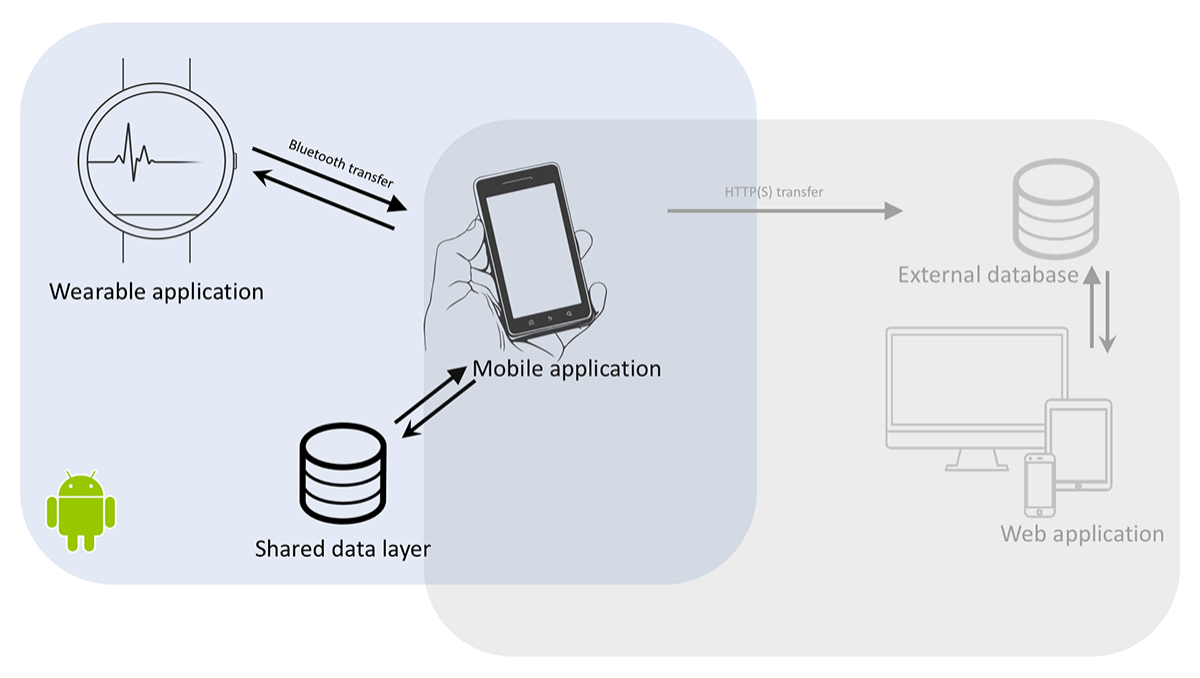
Overview of the setup of the Sense-IT app.

An option to manually export recorded data to a server was also implemented. With this option, a primary user is able to transfer his or her data to a server. These data can then be accessed via an internet browser through a secured gateway. The data can be presented in raw form or plotted to provide a graph depicting the changes in PCEA over time. This option was built in to increase the number of ways the data could be used to support therapy. For example, it could provide an opportunity for the patients to get a better overview of the changes in their arousal over time or to let their therapists have a view (if they were granted access to the secured data by the patient). Although this functionality was built in, it could not be tested at the clinic because of limitations in the technical infrastructure at site and ethical limitations concerning the sending of personal data to servers outside the clinic. Figure 3 gives an overview of the setup of the app.

## Methods

### Study Design

#### The Methodological Framework

**Figure 4:**
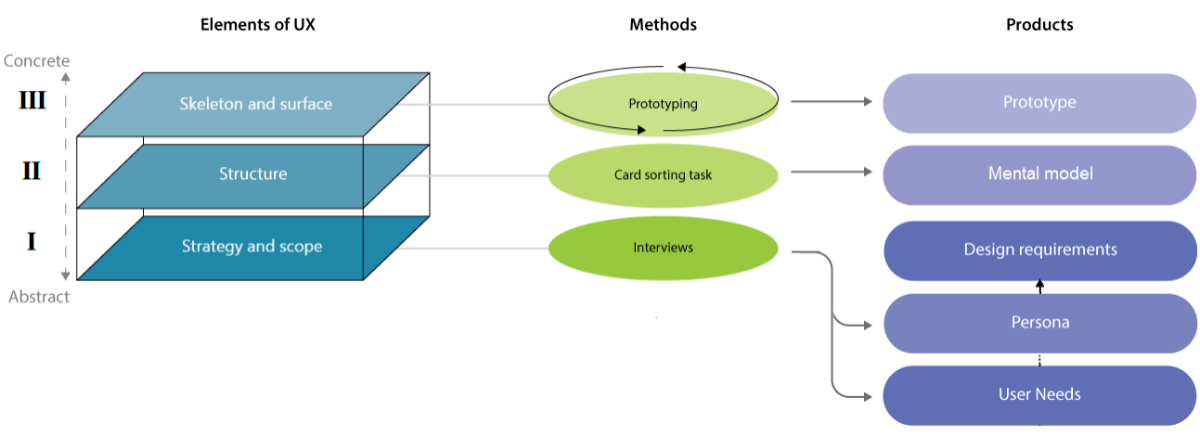
The EMP-framework. The framework is to be read from the bottom (ie, abstract considerations) to the top (ie, concrete considerations). The lines indicate the connection between the elements and the methods. The arrows point to the products that will result from the methods.

A design framework tailored to the setting and purposes was developed based on principles from user experience (UX) design and UCD. We previously published a detailed report on the development of this framework and our first steps in the development of the Sense-IT app [28]. We combined the conceptual overview of *Elements of UX model* [32] with the broader focused approach of the *CeHRes Roadmap* [33] and added a placeholder to specify the methods used. This led to the EMP framework. *EMP* stands for *element-method-product*, the main components of the model. Figure 4 shows a schematic overview of the EMP framework.

We previously completed the steps of the first 2 elements of the framework, that is, the *strategy and scope* and the *structure* plane (in Figure 1, I and II of the elements of UX) [28]. The completion of these steps in the model yielded design requirements and mental models that serve as the basic input for the current, third element of the framework (in Figure 1, III, *skeleton and surface* of the elements of UX). In the third element, the desired product was a working prototype. This implied getting into the back-end organizational structure and flow (*skeleton*) and the front end or outer qualities such as visuals, sounds, and vibrations (*surface*). As the main UX method (the *M* in the EMP framework), we chose cyclic, iterative prototyping [34].

#### Procedure

**Figure 5:**
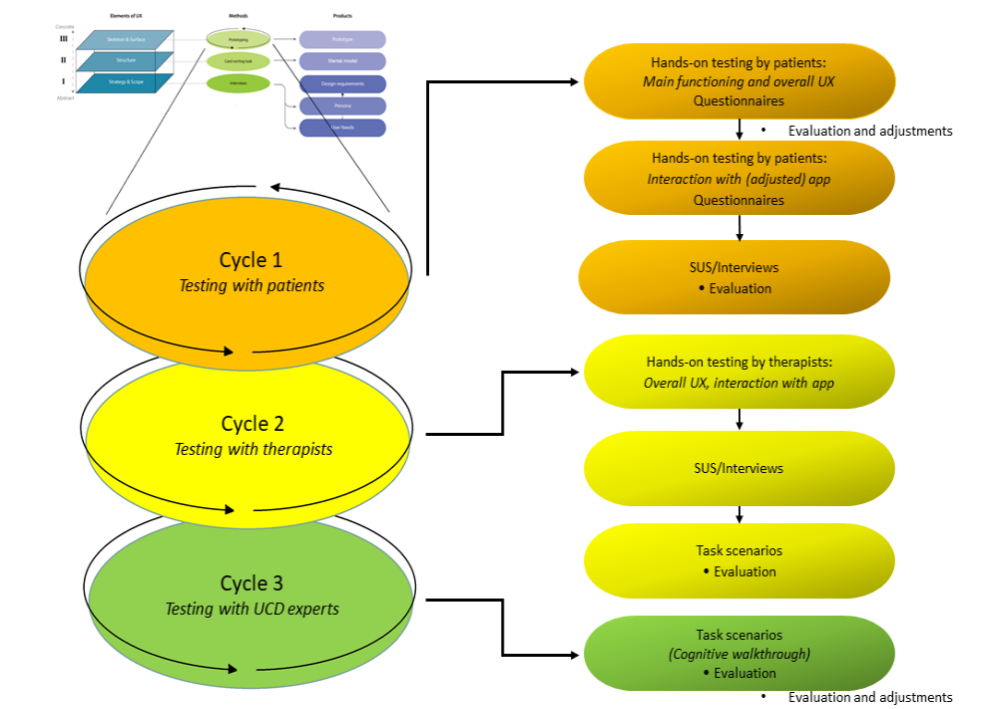
Specification of the Method of Prototyping within the EMP-framework.

We went through 3 cycles of testing, each with its own specific group of users (see Figure 5). The first cycle, that is, initial prototype programming and pilot testing with patients, took place between February and July 2016. The second cycle, that is, usability testing with professional caregivers, took place between November and December 2016. The third cycle, that is, usability testing with expert users, took place between January and February 2017.

### Setting

The first 2 cycles took place within an inpatient psychotherapeutic setting of Scelta, GGNet, a mental health care provider situated in the east of the Netherlands. Testing during the third cycle took place in an office setting within the facilities of the University of Twente.

Scelta is a specialized division within GGNet, one of the larger mental health care providers in the Netherlands. Scelta provides psychiatric treatment of severe personality disorders. At their inpatient clinic, Scelta offers several psychotherapeutic treatment programs for personality disorders. The testing of the app with patients and therapists was within the dialectical behavioral treatment (DBT) [35] unit of the clinic. Here, multidisciplinary group DBT [35] is given to a maximum of 27 inpatients at a time. All patients are diagnosed with 1 or more personality disorders—the majority of them having BPD diagnosed as main disorder. Four days a week, during working hours, patients receive multiple forms of psychological treatment. Medical treatment can be part of the treatment but serves a subsidiary role. On average, duration of treatment within the DBT program is 9 to 12 months. After that, most patients are referred for further (outpatient) care.

### Data Analysis

Data from interviews, questionnaires, and task scenarios were transcribed via a transcription program (*F4*). Additional remarks made by participants during or in between hands-on testing were also registered. Qualitative analysis consisted of a combination of content and thematic analysis [36]. Comments were categorized by applying constant comparison, comparing statements numerically and content wise. Usability problems and needs were listed and subsequently grouped, after which a central theme was allocated to each group. This was done by 2 of the authors (YD and RK), of which one has a background in psychology and the other in computer science. Final categorization reflects consensus after comparison and discussion of the interpretation processes of both authors. The following main themes were identified: (1) technology, (2) user interface and interaction, and (3) functionality (and ethics). Next, within each set of results per group, inductive thematic analysis was used to identify whether, and, if so, which specific values regarding the acceptance and adoption of the app were present. Next to the qualitative analyses, individual and overall scores of the system usability scale (SUS) [37] (used in cycles 1 and 2) and the cognitive walkthrough (used in cycle 3) were quantitatively analyzed.

### Ethical Considerations

The study was granted approval by both a nationwide operating ethical commission and a local ethical commission associated with the university. Participation in the study was on a voluntary basis and only after informed consent. Patients undergoing clinical treatment at the treatment center and therapists working in the center were eligible if they were willing to actively participate. All participants could withdraw from the study at any time with no further obligations. In addition, therapists of the patients held the right to anytime exclude or withdraw patients from participating in the study if they judged a patient’s participation potentially detrimental for his or her well-being or unwanted or inappropriate in any other way. There was no financial reward or other incentive for participating.

### Cycles

As the cycles were carried out as three consecutive studies, each with its own participants, procedure, and materials, the remainder of the method section will be discussed per cycle. Next, results will also be presented per cycle, in paragraphs representing the main themes that resulted from the thematic analysis. Multimedia Appendix 1 pprovides a schematic overview of the thematic ordering. It lists all usability issues and user needs that were identified by 1, 2, or all user groups grouped by the main theme and presents the applied—or planned—solutions and adjustments to the Sense-IT app after the 3 cycles of usability testing. The result section concludes with the presentation of the current version of the Sense-IT app.

### First Cycle: Testing with Patients with Borderline Personality Disorder

#### Participants

A total of 5 patients of the DBT program participated in testing the app. Participation was on a voluntary basis and after informed consent. All were previously diagnosed with BPD and low emotional awareness. Of 5 patients, 4 completed the second iteration, and 1 patient could not participate on the second day of the second iteration, as she forgot that she had to attend other meetings. Patients were aged between 18 and 49 years (mean age 28 years, SD 11.82). All participants were females and all had Dutch as their native language.

#### Procedure

The first cycle consisted of 2 iterations, each with a design phase and an evaluation phase. During the hours of testing, members of the research team remained standby at the clinic in case a patient would encounter technical issues or would come up with questions regarding the app.

During the first iteration, the focus was on the main functioning and overall UX of the app. Patients received a short explanation of the system and some information on the upcoming days. On the first day, patients just had to wear the smartwatch to gather the required HR data to set personal baseline values. They did not have to interact with the system but were asked to monitor their personal experiences on wearing *the hardware*. The second day, patients again wore the equipment running the Sense-IT app. They were asked to use and interact with the Sense-IT app as if it was an actual adjunctive to their therapy. Patients were asked to fill in a questionnaire at the end. After the first iteration, adjustments were made to the app.

During the second iteration, the focus was on patients’ preferences on the (graphical) user interface ([G]UI) and the interaction with the system. It again consisted of 2 days of testing. Patients were instructed to interact with the app as if it was part of their therapy program. The main goal of the second iteration was to examine whether the alterations to the app led to a satisfactory overall UX. At the end of the day, patients were asked to fill in the SUS. They also were briefly interviewed on their experiences.

### Materials

#### Hardware

A total of 5 mobile phones and 5 smartwatches were used in sets. Each set consisted of a Moto G, third-generation smartphone (5-inch screen size and screen resolution 1280×720 pixels) and a Moto 360, second-generation smartwatch (1.37-inch screen size and screen resolution 360×325 pixels). Both ran a version of Android OS: Android 6.x (KitKat) on the mobile phone and Android Wear 1.5 on the smartwatch. Each set was connected via Bluetooth (and optionally via Wi-Fi) through built-in communication software provided by the Android ecosystem.

### System Usability Scale

The SUS is a commonly used questionnaire that quickly and reliably assesses the usability of a product [37-40]. The SUS [39,40] comprised 10 statements that are scored on a 5-point scale, ranging from *totally disagree* to *totally agree*. It contains statements such as “I thought the system was easy to use” and “I thought there was too much inconsistency.” The SUS yields an overall score of the system usability ranging between 0 and 100, where higher scores indicate better usability. For interpretation of scores, we used the guideline provided by Bangor et al [38].

### Questionnaires

A total of 2 short self-constructed questionnaires were administered to the patients during the first cycle of testing. The first questionnaire (26 items) contained questions on the use context; the patient’s level of experience with technology; general UX of the app; UX of the interface on mobile phone and smartwatch; the use of prompts; and additional questions regarding privacy, perceived risks, and missing/desired features. See Multimedia Appendix 2. The second questionnaire (16 items) contained questions on the patient’s general UX with the mobile phone and smartwatch interface, the use of the diary/note keeping function, and questions about the option to share data with therapists in the future. See Multimedia Appendix 3.

#### Interview

After each iteration, a semistructured interview was held with each patient. The interview consisted of 5 open questions. Participants were asked what they liked about the intervention (app plus hardware), what they disliked about it, what their experiences were regarding the measurement of their bodily signals (PCEA), if they would have made other decisions regarding the design of the app and/or choice of the hardware, and if they had any other remarks about what they would like to see improved or altered in a future version.

### Second Cycle: Testing with Therapists

#### Participants

Of 25 health care professionals at the clinic, 4 were invited to participate. They were selected via a stratified sampling method [41]. The sample included 1 psychiatrist, 1 bodily oriented psychotherapist, and 2 groupworkers or sociotherapists. All were native Dutch and aged between 47 and 62 years (mean age 52 years, SD 7.1). Overall, 2 participants were males and 2 were females. One of the participants personally owned a smartwatch. In addition, 3 of the participants indicated to have affinity with wearable technology but indicated not to closely monitor developments in the field of smart devices.

#### Procedure

The app and the 2-day testing procedure were kept similar so that the health care professionals would get a similar UX as the patients. On the second day of testing, a series of 4 paper task scenarios were completed along with the SUS and a short interview.

### Materials

#### Hardware

The same mobile phones and smartwatches were used as in cycle 1.

### System Usability Scale

See the description under cycle 1.

### Task Scenarios

A total of 4 scenarios were written by the research team, based on the functions of the app. Each scenario consisted of a task to be performed on either the mobile phone or smartwatch: (1) *add a comment to your latest measurement*, (2) *change the watch face to another Sense-IT watch face*, (3) *add a general comment to the timeline (not to a measurement)*, and (4) *let the app stop measuring your HR*. With each scenario, the user was asked which steps he or she took to perform the task, what difficulties were encountered while performing the task, and whether the user had further comments or suggestions.

#### Interview

To close off the 2-day hands-on testing phase, a brief, semistructured interview was held with each therapist. The interview questions were the same as in cycle 1, but the interview also inquired how the app could be implemented in the therapies of their patients.

### Third Cycle: Testing with User-Centered Design Experts

#### Participants

For the third cycle, 3 expert users were personally contacted. All were part of the professional network of the authors at the University of Twente. Of 3 expert users, 1 works as an assistant professor in Human Centred Embodied Design, with expertise in the field of assistive technologies. The second expert user is a researcher with a background in Biomedical Engineering who currently conducts research on telemonitoring in a medical setting. The third is an assistant professor in Product Interaction Design whose current work is focused on multisensory design, user interaction and experience, and their influence on behavior and motivation.

#### Procedure

The UCD experts were asked to complete a cognitive walkthrough while using the app in an individual session. They were provided with information on the primary users and their concerns in the form of a persona [28]. After the cognitive walkthrough, each UCD expert was given a final moment to reflect and mention any other detected usability problem they had encountered.

### Materials

#### Hardware

The same mobile phones and smartwatches were used as in cycles 1 and 2.

### Cognitive Walkthrough

The UCD experts were asked to complete several tasks while thinking out loud about what a primary user (ie, a patient with BPD and low emotional awareness) would do and evaluate if the task at hand would be easily achievable. The tasks were the same as the scenarios for the therapists.

There were 4 additional questions on usability to be answered with *yes* or *no*. These questions were as follows: (1) *Will the primary user try to achieve the correct effect?*, (2) *Will the primary user notice that the correct action is available?*, (3) *Will the primary user associate the correct action with the desired effect?*, and, if the user performed the right action, (4) *Will the primary user notice that progress is being made toward accomplishment of her goal?* As an indication of usability error [41], the average number of *yes* answers were added up to a score between 0 and 1, with higher scores indicating less errors.

## Results

### First Cycle Results

#### Quantitative Data (System Usability Scale)

The patients rated the system after the second iteration of the first cycle. The overall rating was *good* (average score of 78.8). Of 4 participants, 3 rated the app as good to *excellent* (85 to 97.5), whereas one rated it as *poor* (42.5) [38]. The latter participant experienced trouble in working with the mobile phone and smartwatch in general and indicated that her age could play a role. She would therefore have liked to see the user interface simplified and more *foolproof*. She also would have liked to have an easy to understand user manual to come with the app.

#### Qualitative Data (Questionnaires and Interview)

#### Technology

Overall, patients were positive about the ease of use of the app on the smartwatch and mobile phone. They also liked the design and indicated they had no problems with having to wear a smartwatch—although not all of them were used to wearing a watch. Moreover, 3 patients found it cumbersome that the app was on a mobile phone of the project, so they had to carry it besides their own device. Given the test phase, all understood why the app was not installed on their own devices.

### User Interface and Interaction

All patients experienced the app as discreet and inconspicuous. All would have liked more data visualization. They stressed it was important that the data visualization should remain *neutral* for reasons of discreetness while still being intuitively understandable. Regarding the Watch face GUI, users found it important that they could choose from several designs.

Patients stressed that the app should be compatible with their therapies. This need was already addressed, as physiological arousal was represented in a *two times five* stage process that is compatible with the current user setting (DBT and systems training for emotional predictability and problem solving [3,42]) as well as cognitive behavior therapy (CBT). Patients indicated that other ways of breaking down in levels would still be welcome for the use of the app in other forms of therapy. One of the patients believed that the levels of the physiological scale were representing the subjective emotional states that were used in the main model on emotions in therapy.

The timing and frequency of feedback given by the app (via the vibration motor in the smartwatch) yielded mixed reactions. One patient said the notifications matched the perceived moments of arousal well. For 2 patients, the frequency of notifications was acceptable. One patient received *a bit too many notifications*, especially since they were accompanied by a vibration of the smartwatch that was noticed by other patients during group therapy. In contrast, another patient received too little notifications in regard to her experienced moments of heightened emotional arousal. She also believed the notifications were sometimes lagging behind with her actual feeling of increases in arousal. The reliability of the app was perceived as average to slightly unreliable. One patient encountered a problem in HR measurement, which was discovered and adjusted the second day.

#### Functionality (and Ethics)

One of the main findings after the first iteration with patients was the expressed need for an option to add a note to a registered change in PCEA level by the app. Regarding privacy, all patients answered that future sharing of the information generated by the app with their therapists would not be an issue for them.

### Adjustments and New Features Added After the First Iteration

To prevent confusion of *actual*, subjective emotional arousal and the PCEA provided by the Sense-IT app, the numerical denoting of the scales was changed to icons consisting of spheres. Here, 1 sphere is equivalent to level 1, and 5 spheres are equivalent to level 5. One watch face was replaced by a new one, as the original one was judged as little attractive.

Next to adjustments in graphical layout, the app was extended with 4 new features. Of 4 features, 2 were based on the feedback received from the primary users, and the other 2 were based on the review of the app by the research team. The first new feature was the option for the user to provide feedback to the system by adding personal notes to each notification of change in PCEA by the Sense-IT app.

The second new feature was the option to add personal notes to a *diary* that is unrelated to recorded changes in PCEA level. As this is a common element in regular psychological treatments such as CBT, the idea for adding a diary was to enable patients to comment on their experiences over longer periods of time, for example, their morning, whole day, week, instead of adding comments to momentary situations captured in the app. This is also in line with recommendations from a recent review study of mobile health (mHealth) mobile phone apps [43]. Both features were added in response to an expressed need for a memory aid. Such an aid could be used, for example, during appointments with professional caregivers.

The third feature was a settings tab in which several personal values/preferences could be set. The addition of this feature was based on a review of the app by the research team. It concerned the option to manually adjust the mean HR and SD used by the system, as well as an option to alter the *sensitivity* of the system, that is, decrease or increase the threshold of when a *new level* was reached. We implemented 3 levels of sensitivity: *normal* (every change in HR of 1 SD adds or subtracts a PCEA level), *low* (change of 1.5 SD), and *high* (change of 0.5 SD). The option to set the sensitivity of the app was implemented to better adjust to the preferred level of feedback received by the user. The option to manually alter the values of mean HR and SD was primarily added to let the researchers override the values set by the system based on the baseline measurement in case the set values were unrepresentative for the user (eg, the user turned out to have an uncommonly low or high average HR during baseline measurement). The perceived *lagging* of feedback that 1 primary user reported could not be addressed in this stage of development but was scheduled as future work.

**Figure 6:**
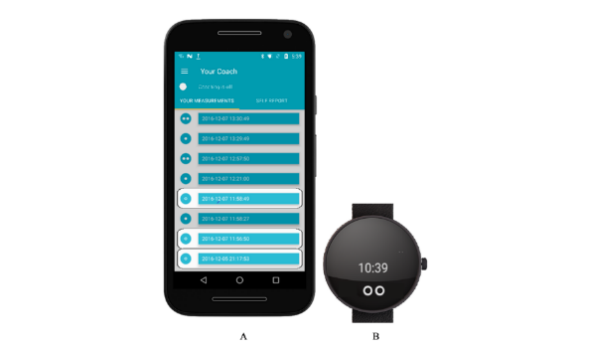
Examples illustrating the visualization of the low-arousal state “hollow spheres” on the smartphone (A) and smartwatch (B) interface.

A fourth new feature was the ability of the app to also measure and represent PCEA states up to (theoretically) 5 steps below the user’s average HR. The decision to add this feature was made by the research team and was based on current expert theory on emotional (under)arousal in the field of affective neuroscience and psychotherapy [44]. These states were visualized in the app as hollow and/or blue-colored spheres (see Figure 6).

### Second Iteration

The adjusted version of the app was used in the second iteration. All patients indicated that they experienced the option to add notes to detected changes in PCEA as useful. Of 5 patients, 3 had used the feature on more than 1 occasion. The diary for miscellaneous notes was also perceived as useful by all patients, although none of them had actually used this function.

The representation of PCEA states with below the personal baseline was also regarded as useful: “By adding this feature, the therapeutic goal to reach or maintain an overall lower state of arousal is supported by the app.” Patients suggested that they could practice mindfulness or do an exercise from their relaxation training and see if it had a lowering effect on their level of arousal.

All patients indicated that the use of notifications increased their self-awareness. Although 2 of them indicated that the app initially induced some more stress, for example, by making them become too focused on the feedback from the app and reacting to that, all indicated they quickly got used to *being monitored continuously*. All indicated they would like to see this app being integrated into their therapy, preferably as an adjunct that could be used in regular face-to-face meetings with their therapists. They all thought the app had positively affected their awareness of their emotions. One patient indicated she got notifications about increased physiological arousal during group therapy, which led her to observe her emotional arousal more closely. She thought this had really helped her to make more out of the session. All participants answered they would have liked to continue wearing the devices and using the app all day.

### Second Cycle Results

#### Quantitative Data (System Usability Scale)

The therapists rated the system with SUS scores between 30 and 85, with a mean of 59.4, which is *OK* for the usability of the app, but *a candidate for increased scrutiny and continued improvement* for passable products [38].

All therapists described the app as useful. The lowest SUS score was because of a reliability issue, as she did not receive any notification during testing. However, she still expressed a positive attitude toward the app: “The app did not function properly, so that’s a main reason why I reported negative experiences [on the SUS; authors]. However, I still welcome the therapeutic function the app could fulfill, so keep up the good work!”

#### Qualitative Results (Task Scenarios and Interview)

Overall, the feedback by the therapists conveyed as a general message that the app bears real potential but should first be disposed of all bugs and errors. They all stressed how important it was the app should not attract *unwanted attention* in any way when integrated in daily practice, either meaning it should not hinder their daily work as a therapist (eg, having to do *extra work* when the app should start malfunctioning) nor disrupt standard forms of therapy (eg, a notification by the app draws the patient’s attention, which disrupts the process of face-to-face therapy) or draw unwanted attention to the patient in social settings.

#### Technology

The therapists mentioned that every now and then the system seemed to stop processing HR data. Overall, 2 said such technical difficulties were experienced as highly demotivating and could cause stress with patients. In addition, 3 therapists mentioned that it was unclear to them how far apart the smartwatch and mobile phone can be without losing the Bluetooth connection, which caused a feeling of uncertainty. One suggested to add a manual (in the app or just on paper) that provides such information. One also suggested that it would be better to have multiple sources of physiological data, as this could improve the accuracy of the PCEA.

### User Interface and Interaction

The general layout of various buttons proved less then optimally intuitive for 2 of the 4 therapists. During the scenarios, they found the option to add a miscellaneous note confusing when asked to add a comment to the latest registered change in PCEA. All indicated that the *self-report* tab was easy to find and leaving a note was easy to do. Also, ending the measurement of HR was not clear to the 2 previously mentioned therapists. Although the other 2 immediately correctly pressed the yellow switch to stop the measurement, 1 of the other 2 did not manage to find the on/off switch at all.

Furthermore, 2 of the therapists found the graphical representation in spheres convenient and logical. The other 2 therapists, however, did not figure out the meaning of the spheres by themselves. In addition, for both these therapists, it was not clear what the buzzing of the device intended to convey. They did understand how the app worked after it was explained to them by the researchers. One of the 2 therapists who understood the app *by intuition* commented that the app should be even more *graphical*: it should present the user with more graphs and figures.

#### Functionality (and Ethics)

All therapists mentioned it was of significant clinical relevance that the app communicated not only states of PCEA that were higher than the primary user’s baseline but also those that were below the personal mean. This way, patients can comment on their *physiologically calm* moments as well and learn from it. None of the professionals was able to change the interface of the smartwatch. The set of operations required by the user proved to be nonintuitive and prone to errors, resulting in the app to stop working.

### Third Cycle Results

Overall, UCD experts considered it to be easy for users to access information and perform actions but also gave several suggestions for improvement of the app.

#### Quantitative Data (Cognitive Walkthrough Evaluation)

The average scores per task on the cognitive walkthrough ranged between 0.83 and 1.00 and the average score across tasks was 0.87. These scores indicate that the app’s overall usability is somewhere between “OK” and “acceptable,” yet could be improved.

#### Qualitative Data (Cognitive Walkthrough Evaluation)

#### Technology

A problem indicated by all 3 experts was that the collecting and processing of sensor data by the app seemed to stop at random moments, although they were not certain if this was really the case. When the Sense-IT app is turned off within the mobile phone environment, the user sees a message on the smartwatch that says the app is waiting to receive data from the sensors. This was judged as confusing to the user, as it does not specify whether the app is idle and awaiting an action by the user or it is waiting for sensor data that will be transferred automatically.

What was found missing was an electronic or paper manual. One of the UCD experts indicated that not all functions/features of the app are intuitively clear to the user. He suggested to include a manual with information on how the app works, what the different number of circles mean, and what a user can do with this information.

### User Interface and Interaction

#### Navigation

Overall, the experts judged the interface to be low in complexity and easy to comprehend. Working with the app was judged as simple, and most tasks were easy to perform. Still, navigation within the app could be improved. Several small flaws negatively influenced the flow and experience of the interaction. Changing the interface of the app on the smartwatch was judged as intuitive, however, *only* when users are familiar with using a smartwatch or mobile phone. The option to go back to the previous screen was sometimes hard to find. The location of the on/off switch could be better. As people typically search for an on/off switch on the upper (right) corner of a device (such as a remote or a mobile phone), it was advised to place it there in the app as well. It was considered an additional issue that the smartwatch did not give visual or haptic feedback to the user when the collection of sensor data stopped.

All 3 UCD experts considered it easy to figure out how to add a comment to the latest registered change in PCEA. For added clarity, the text *latest measurement* could be added on top of the column. One expert suggested to link the *self-report* function on the main screen of the app to a button, not a separate tab. All expressed doubts about the usefulness of the option to add miscellaneous notes apart from adding notes to detected changes in PCEA. It was suggested that the functionality and UX could be improved by integrating both in 1 timeline.

#### Visual Layout

Another navigational issue concerns the visual layout of the homepage. When the user is on 1 page, the other page/tab is displayed in light gray shading. This might suggest that this function is not available. On the opposite, the color of the text *coaching is on/off* is the same as the active buttons, which suggests it can be switched on and off by touching it, while it cannot. The experts suggested that a change in colors or layout can solve this inconsistency.

#### Textual Layout

Next, some windows or buttons contained words that were too *psychological* or *technical* and thus were difficult to understand for most users. Examples were the line of text “You are high in your physiological scale” and the term *self-report*.

#### Personalization

The UCD experts made several suggestions to enhance the level of personalization within the app. In the settings menu, a user identification is displayed in the current version of the app with a number that does not mean anything to the user. It was suggested to replace this number with the option for users to add their own name. Other suggestions were adding options to switch contrast of the GUI (black/white conversion) and/or the option for the user to personally set the colors or choose between several color schemes.

#### Persuasiveness

The experts gave several suggestions to increase persuasiveness. A suggestion to persuade the user to add notes and comments was to include changing colors (a detected change with a note gets another color than one without) or by displaying a commenting space with each detected change in PCEA, so it is directly visible where personal notes have (not yet) been added. It was also suggested to include on-screen notifications on the mobile phone and visual reminders on the smartwatch. When receiving an on-screen notification, a comment could then be added without having to open the actual app. The experts suggested to have the app actively request to add a comment at more levels of PCEA. In the tested version, the app only does so when the user’s PCEA reaches the fourth or fifth state. One expert advised to further integrate the use of emoticons. At the moment, the app already allows the user to add emoticons to a note, but a suggestion was to add a separate column for emoticons in the notes section.

#### Visualization of Measurements

Several suggestions for improvement were about presenting the user a more *graphical* overview. In the tested version of the app, the overview of all measurements is fairly textual. To improve the overview for the user, colors could be added such as, for example, darker blue on the higher emotions. Also, a graphical timeline with a scalable timeframe (day, week, and month) could be implemented to track the PCEA.

#### Functionality (and Ethics)

A suggestion was to add the option to take pictures or record videos via the camera of the mobile phone. In the settings menu, not all options were clear to the UCD experts.

### Sense-IT: Current App

On the basis of the analysis of the identified usability problems and user needs (and proposed solutions) gathered after completing all 3 cycles, the Sense-IT app was revised. The app as a whole still consists of a wearable app on a smartwatch and a mobile app on a mobile phone, all implemented using the Android ecosystem. Below, we describe its components and functionalities.

#### The Mobile Phone App

The mobile phone app of the Sense-IT system consists of 3 components: (1) communication and storing of data from the wearable app, (2) the algorithm detecting changes in physiological arousal, and (3) the user interface.

The *first* component reads the data pushed from the wearable app and stores the data in a local database on the mobile phone itself. The algorithm then evaluates changes of PCEA based on new available data. By default, new and old data are compared every 10 seconds. The time between evaluations can be altered (limited by the specifications of the hardware).

The *second* component is the algorithm and takes into account the personal average HR and SD of the user and the current activity and the current HR of the user. It classifies the PCEA to 1 of 10 levels ranging from −5 to −1 and from 1 to 5. The app notifies users when their HR (measured via PPG) decreases or increases markedly (the boundaries are determined by the user’s mean HR and SD, but these values can be personalized). The average HR and SD are based on the results of a baseline measurement in which HR is measured until a preset number of valid measurements (standard setting is 300 measurements) is collected. Notified changes will be mostly unrelated to physical activity, as the app will only notify the user when he or she is not involved in vigorous action (as determined by the onboard accelerometer and associated activity recognition algorithms). All changes in PCEA, together with the classified type of user activity, are recorded and displayed in the overview of the mobile phone app.

**Figure 7:**
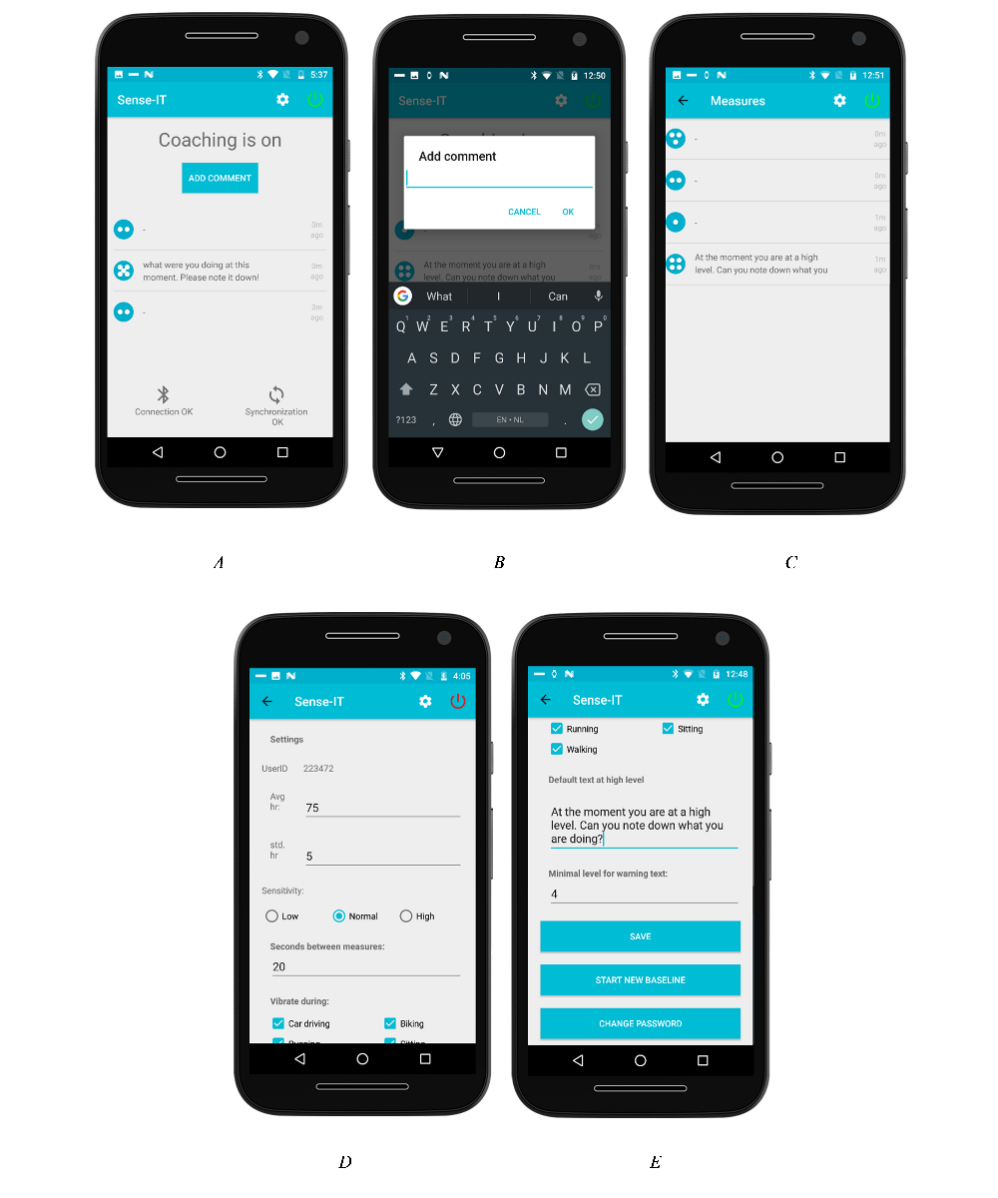
UI of the current version of the Sense-IT smartphone app (A-E).

The *third* component, the user interface, supports interaction between user and app. The dashboard page of the app (Figure 7A) presents an overview of the status of the app, for example, the status of the connections and synchronization. The last 3 detected changes in arousal are displayed, and there is an option to add notes. Clicking it brings up a new window where notes can be added (Figure 7B). Users can turn on the app by pressing the on/off icon in the top-right corner of the user interface. By clicking on *show more* (which appears in a box below the last 3 detected changes once there are more than 3 recorded changes), the user opens a timeline of all changes detected by the system (Figure 7C). The events are listed in chronological order. The events in the timeline are displayed with their level, the message written by the user, and the time when the event did happen. By clicking on one of the events, the user can add a note or edit a note that was stored (see Figure 7B). The (scrollable) settings page of the app is opened by clicking the settings icon on the dashboard (Figure 7D). To prevent changes in the settings by unauthorized users, this page is password protected. Within the settings menu, the user can (re)start a new baseline measurement (see Figure 7E). Measured values can also be manually adjusted if needed. Other adjustable settings include the sensitivity of the algorithm (high-medium-low), the time by which the algorithm checks for changes in physiological arousal, and the option to select the type of activities whereby the app should or should not give a notification when a change of physiological arousal is detected (see Figure 7D). Users can define their own message that will be displayed when their PCEA reaches a predefined level. This level can also be set by users in the setting page (Figure 7E, both options are in the middle section of the screen).

#### The Wearable App

The wearable app uses the accelerometer (movement) and PPG (HR) sensors on the smartwatch to monitor the user. Sensor events are registered as fast as possible by the hardware via the Android sensor manager API and registered on the smartwatch app. With the hardware used in this study, this comes down to once per second. A sensor event contains data fields associated with the event. In the case of an HR registration, these data fields include the sensor that generated the event, the accuracy of the event, the timestamp of the event, and the value of the event in beats per minute. The accuracy value is a value between −1 (no contact) and 3 (most accurate) that is determined by Wear OS. The Sense-IT app is currently set to include all measurements with value 1 and higher. These measurements are sent through the device APIs as data events and are received by the mobile phone for further processing.

**Figure 8:**
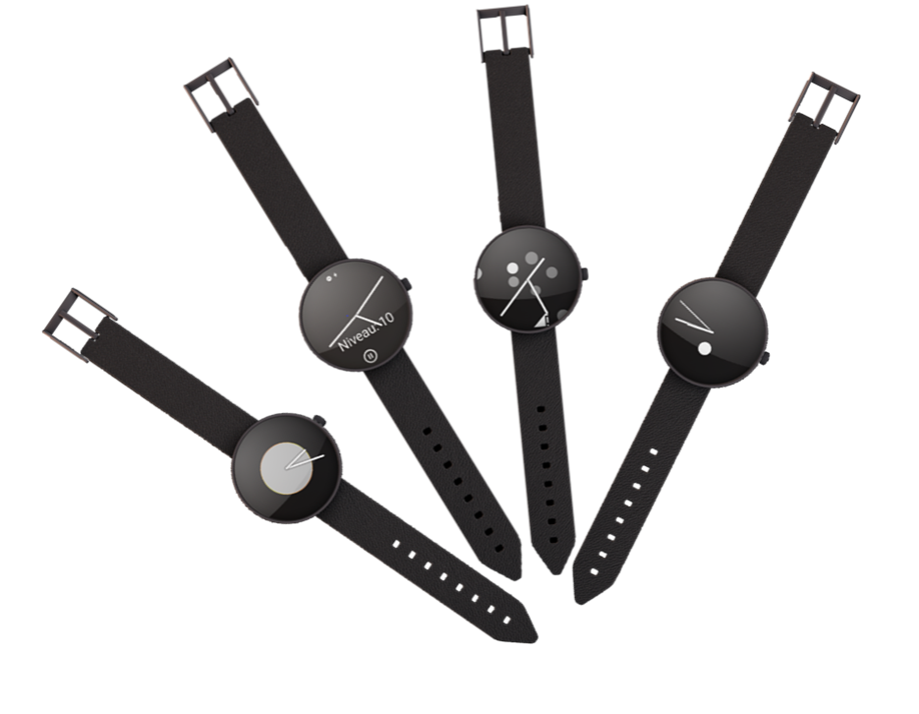
Current watch faces.

The wearable app is also responsible for presenting the GUI on the smartwatch. On the basis of data from the sensors and the result of the algorithm of the app on the mobile phone, the screen of the watch will present the current state of the measurements via 1 of the 4 available watch faces. Figure 8 shows the current watch faces. The user can alternate between them and choose the one they like via the Wear OS settings.

## Discussion

### Primary Findings

This paper describes the development and usability testing of Sense-IT, a wearable biofeedback app for Android-based devices that can be used in the daily practice of a mental health clinic for personality disorders. The app is meant to support BPD patients in increasing their level of emotional awareness. The app was tested on usability by patients, therapists, and UCD experts.

The concept of a biosensor-informed app for emotional awareness enhancement was appreciated by both patients and therapists; the prototype was judged as promising by all user groups. After 1 iteration in the first cycle of testing with patients, basic functionality of the Sense-IT system was rated as acceptable. This waived the need for a major revision before starting the consecutive rounds of testing with mental health care professionals and UCD experts. Still, results after finishing all 3 testing cycles made clear that the app should be considered a *candidate for increased scrutiny and continued improvement* [38]. In total, 30 usability problems and/or needs were identified (Multimedia Appendix 1). All 3 user groups brought up usability problems and suggestions for improvement. UCD experts identified most of these usability problems (20 in total). Patients identified 11 issues, and therapists identified 9 issues. There was some overlap between them: 8 issues were mentioned by at least 2 groups. The UCD experts brought to our attention 14 themes or problems that were not mentioned by the other groups, the patients 6, and the therapists 2. Most usability problems and needs could be addressed in the software revision that followed after the 3 cycles were finished. This resulted in the version of the Sense-IT app presented at the end of the results section.

Results of the 3 cycles of testing favor the use of ambulatory biofeedback to improve emotional awareness in patients suffering from BPD and low emotional awareness. To our knowledge, the Sense-IT is one of the first scientifically grounded apps that can be used in clinical research and/or clinical settings for longer periods of time without requiring extensive support by researchers and/or developers. Although it may be a quite simple app from a technological perspective, from a mental health perspective, it is a real innovation. This applies to both the way it was developed as to it being a new way of delivering treatment to patients. The use of consumer technology to enable *always available* indices of physiological changes could prove to be a relevant addition to existing therapeutic interventions, even if the measurements and algorithms used are relatively crude and simple, and the integrated sensors have limited resolution and less than perfect accuracy. There is an ever-growing number of publications that introduce and discuss concepts of biosensor-informed mHealth interventions on emotional/psychological awareness [45-47]. However, at present, *feeling the changes in the body* is something patients with mental health problems still mainly have to learn by purely subjective methods. We found 1 recent project in which—nonwearable—technology was used in a mental health setting that aimed to “direct alexithymic persons to reflect on their internal, somatic experiences as a source of information for interpreting and labeling emotional experiences” [15]. Although there are numerous commercial companies and startups that sell products and/or apps that claim to help to raise awareness on—or even directly measure—emotion, emotional arousal, or stress, they generally lack research that validates or supports their relevance for users and/or validity (see the study by Peake et al [48] for just a select number of examples).

### Limitations and Strengths

Of course, both the app and the study have their limitations. Regarding the choice of hardware, research-oriented hardware that gives access to more advanced or potentially *better* sources of physiological data such as EDA and/or HRV exists [49]. However, the use of HR as the main cardiovascular parameter for physiological arousal is a defendable option. HR, such as HRV and EDA, is triggered by the sympathetic nervous system [29,30]. Typical consumer wrist-based HR monitors provide a fairly accurate measurement of HR even when deployed during physical activity and movement [31]. Measuring HRV is much more susceptible for producing artifacts under real-world conditions. Even producers of wearable technology such as the E4, which is claimed to be able to measure HRV from PPG, stress that this is only feasible in short scenarios (ie, several minutes) that are free of movement.

We believe that with time, better (consumer) hardware with more and more advanced sensors will become available, as will be the case for more accurate signal processing algorithms that can be used in (validated) wearable devices [50]. Improving hardware or data processing algorithms is not what is at the core of our project. However, the Sense-IT is first in providing a new, stable platform that can be considered a *new type of intervention in mental health practice*.

Regarding the OS for which the app was built, the app is currently only available on devices running Android and Wear OS. We did not develop a version for any other OS, such as iOS, because in this study, the hardware was provided to the participants. In addition, Android roughly has had a 75% market share worldwide over the last years in contrast to 22% market share for iOS [51].

More participants could have been included to ensure saturation of feedback and overall group representativeness. To gather a relatively high number of relevant remarks and comments without too many duplicates, we included 3 to 5 users per group per iteration. On the basis of the literature, these numbers seem proportionate, although more could have been better [52,53].

Regarding the selected use case scenario, it could be considered a limitation of this study that it is exclusively focused on the use of the app with patients with BPD *and* low emotional awareness. Of course, use of the app by other patient and nonpatient groups with low emotional awareness seems feasible after context-dependent tailoring [54]. However, if an app works for one of the most challenging groups of users in terms of emotional regulation, it could very well work well for others too [55]. Since the start of the project, researchers from 2 other Dutch health care institutions have joined our group and set up studies with the Sense-IT app within their own settings and their specific patient groups. These studies concern the usefulness, usability, and effect on clinical outcome measures of Sense-IT for patients in forensic psychiatric care with aggression regulation problems, and for adolescents in residential care who have many conflicts because they struggle to detect increasing levels of stress.

Considerable effort was made to ensure *trustworthiness* of this qualitative research project—as, for example, discussed by Shenton [56]. We believe this to be one of the strengths of this study. Shenton mentions 4 criteria for trustworthiness that were originally formulated by Guba for assessing trustworthiness in naturalistic inquiries [57]: credibility (in preference to internal validity), transferability (in preference to external validity/generalizability), dependability (in preference to reliability), and confirmability (in preference to objectivity). To ensure *credibility*, we adopted a *design science* paradigm [58,59] to construct and simultaneously test a scientifically informed approach in designing an mHealth app, using well-recognized research methods. We developed early familiarity with the setting, patients, and therapists. We used different methods and different types of informants. We stimulated honesty in interviewing the participants and used UCD experts to also assess the app. We previously published a detailed report on how this was done and provided a description of the backgrounds, qualifications, and experience of the researchers [28]. To ensure *transferability*, we have provided ample background data to establish the context of study and gave a detailed description of the phenomenon of interest. As mentioned in this section, we started collaborations with other researchers from other settings to study the app in different environments. To ensure *dependability*, we applied several overlapping methods and used an iterative design approach when testing with the patients in which the results of the second cycle served as a *test* of the correct interpretation of the results of the first one. In addition, with the introduction of the EMP framework, we delivered an in-depth methodological description that should allow others to repeat our study. *Confirmability* should be evident from this and previous publication, in which our work was put up for thorough peer review.

What this study added to the literature is an example of how development of an mHealth app within a clinical mental health setting can be challenging, yet feasible, and that it can result in a stable working prototype of an app. It also shows how the use of the multiple user groups is of added value during design and realization. In this study, the app was not yet tested for clinical effectiveness. Although this is perhaps not so much a limitation of the study, as the usability and stability of the app should be tested before using and testing it as a clinical intervention, it is a question that has to be addressed before use of the app can really be recommended for use as an adjunct in psychotherapies. Such is planned later this year in the same setting as this study took place. Furthermore, a second, graphically more advanced GUI for Sense-IT is currently developed by a dedicated graphic/UX designer to further optimize the UX.

### Conclusions

In this study, mHealth development within a clinical mental health setting proved to be challenging, yet feasible and welcomed by targeted users. The Sense-IT app was met with enthusiasm and openness by both patients with BPD and therapists, groups that are both known to be reluctant to embrace technological innovations. The use of the EMP framework and the involvement of multiple user groups proved to be of added value during design and realization, as evidenced by the complementary requirements and perspectives. If the app proves to be effective after further clinical testing, Sense-IT would be one of the first broadly applicable technological interventions in the treatment of BPD—and probably in general mental health care—that is actually *new* as it is not the next form of *talking cure* (ie, psychotherapy), medical treatment, or traditional skills or behavioral training. It would support the treatment of BPD by directly addressing one of the most important factors in BPD, namely limited emotional awareness [6]. In general, it could enable patients to take *therapy* out of the therapist’s office into their lives far easier.

## Appendix

Multimedia Appendix 1 Overview of all usability problems and/or needs that were identified.

Multimedia Appendix 2 Questionnaire used with the patients after the first iteration.

Multimedia Appendix 3 Questionnaire used with the patients after the second iteration.

## Abbreviations

API: application programming interface
BPD: borderline personality disorder
CBT: cognitive behavior therapy
DBT: dialectical behavioral treatment
EDA: electrodermal activity
GUI: graphical user interface
HR: heart rate
HRV: heart rate variability
mHealth: mobile health
OS: operating system
PCEA: physiological correlate of emotional arousal
PPG: photoplethysmogram
SUS: system usability scale
UCD: user-centered design
UX: user experience
