# Supplementary material for: Development of an Ambulatory Biofeedback App to Enhance Emotional Awareness in Patients with Borderline Personality Disorder: Multicycle Usability Testing Study
Source: JMIR Mhealth Uhealth. 2019 Oct 15;7(10):e13479. doi: 10.2196/13479 (PMC6913718; doi:10.2196/13479)
Supplement: Multimedia Appendix 1 [file mhealth_v7i10e13479_app1.pdf]

Theme	Usability problem / Need	Mentioned by (number)	Response / Solution	Unsolved / Planned future work
Technology	Process of monitoring HR ends at seemingly random moments after some time of functioning. Smartphone and smartwatch lose connection. Stability is essential for a smooth user experience and adherence.	Primary users (3)<br>Secondary users (4)<br>Expert Users (3)	Improved overall stability by reprogramming components checking by external software company. A monitoring function was added so the user could check whether there is an active connection between devices and whether bio sensor data is being sent/received.	
Technology	For better validity and reliability, input via multiple (biosensor) sources is desirable	Secondary users (1)	Via a native Android algorithm, data from gyroscope and accelerometer is now used to determine several states of physical movement/activity when not sitting, such as walking, running, riding a bike. This information is displayed with each detected change in PCEA in the overview of the smartphone app. This can help determine the nature of the change in PCEA, but also help to clarify/remember the context in which the change occurred	*Future work:* Enabling input and processing of other sources of (physiological) data, such as EDA, HRV, voice-analysis, et cetera.
Technology: *Manual*	Basic functioning (such as the vibrating signal of the smartwatch), how to use and interact with the app is not clear to users without proper explanation of the app.<br>Suggestion to include an e-manual inside the app or a regular paper manual.	Secondary users (4)<br>Expert users (1)	Paper manual explaining basic functioning of the app (i.e. meaning of the vibration signal on the watch) and the hardware it is used on (i.e. range of Bluetooth signal). This was chosen over an e-manual for reasons of low visual complexity and keeping an clean and simple interface for an optimal UX for this user group (see our previous paper on the preferred flow and structure of the app)	
User interface and interaction	Neutral yet intuitive visuals in GUI is essential	Primary users (5),	Adhered to the 'Material Design' lay-out by Google	
User interface and interaction	GUI should be compatible with multiple theoretical frameworks on emotional arousal	Primary users (5)	The option to choose between partitioning of the PCEA level between level -5 /+5, levels 1 to 10, -3 to +3	*Future work:* Option to manually set the number of levels of PCEA represented by the system
User interface and interaction	There can be confusion about the difference between emotional arousal and PCEA	Primary users (1)	Visualization of level of PCEA with spheres instead of numbers	
User interface and interaction	The navigation/ basic lay-out within the app could be improved	Primary users (1)<br>Expert users (3)	Gave the whole smartphone app a new look: Simplified lay-out, a clean and clear color scheme, lesser text on screen, use of less 'psychological' terminology, more clear, generic symbols as buttons for different functions, repositioning of buttons according to received feedback, one main screen instead of tabs, clear back-buttons in sub-screens	
User interface and interaction	Difference between adding a comment to registered change in PCEA and adding a miscellaneous comment is not clear	Secondary users (2)	Implemented different symbols for both types of entries	
User interface and interaction	Button to end HR-measuring is not easy to find, suggestion by expert to place it on top of the screen in the right corner	Secondary users (2)<br>Expert users (1)	Placement of the button on the upper right corner of screen of the smartphone app; changing of symbol from a flip-on/flip-off switch to a on/off-button. Changes intend to let the screen lay-out more resemble a generic remote used with a tv or stereo.<br>We also added a centrally placed text in capitals, indicating either 'YOUR COACH IS OFF' ('JOUW COACH STAAT UIT') or 'YOUR COACH IS ON' ('JOUW COACH STAAT AAN')	
User interface and interaction	Purpose of the separate diary in which the user can add miscellaneous notes may not be clear to the user: placed under a separate tab/ on separate page it will not be used (often). Suggestion to include this functionality on the main screen by adding a single button	Secondary users (2)<br>Expert users (3)	The smartphone app now has a centrally placed button in the upper half of the main screen to add notes	
User interface and interaction	In the overview on the main screen of the smartphone app, it may not be immediately clear to the user what are the latest detected changes in PCEA.	Expert Users (1)	Current version of Sense-IT only shows the three last changes on the main screen, annotation of time of detected change in PCEA is changed to 'time past since measurement X was done'.	
User interface and interaction	There is quite a lot text on the main screen, some texts are potentially unclear or even confusing to the user (too 'psychological')	Expert Users (2)	Amount of text and numbers has been reduced current version for a cleaner look, text has been simplified and/or replaced by symbols.	
User interface and interaction	Increase visual clarity of the smartphone GUI: make each line look more integrated and thus clear to read	Expert Users (1)	Increased on screen distance between each detected change in PCEA and addition of gray horizontal lines between each measurement.	
User interface and interaction	The name of the option to add miscellaneous notes, 'Self report' ('zelfrapportage'), is a term that is too 'psychological' for an average primary user.	Expert users (2)	The text on the newly added button is changed to 'add a comment' ('notitie toevoegen').	
User interface and interaction	Suggestion to integrate both types of input in one timeline	Expert users (1)	Both types of input (detected changes in PCEA with or without comment, and miscellaneous, single comments) now appear in the same timeline.	

User interface and interaction	The distinction between a miscellaneous note and a note added to a detected change in PCEA is not sufficiently clear to the user	Expert users (2)	Clear symbols for each type of entry to easily differentiate between the two on the timeline.	
User interface and interaction: *Persuasiveness*	Increase persuasiveness to add a note by adding a commenting space to each measurement which has not yet been filled in.	Expert users (1)	Each detected change in PCEA now has a space which contains (part of) the note added by the user, or a '-' to remind the user they did not add a note (yet).	
User interface and interaction: *Persuasiveness*	Adding the circles on the comment section could serve the user as a reminder of the level of PCEA to which he/she is adding a note	Expert users (1)		Not yet implemented
User interface and interaction: *Persuasiveness*	Increase persuasiveness of the app by adding on screen 'reminders' to add notes	Expert users (1)		Not implemented, since there is not yet any evidence the app should be that more persuasive: main purpose is to increase awareness. It has to be determined whether increased persuasion to add notes serves this purpose in the given user scenario. Moreover, unobtrusiveness and inconspicuousness were identified as main user requirements. Adding features as visual reminders interferes and jeopardizes the work done to ensure these requirements are met in the design.
Functionality *(basic)*	Recognizing lower states of physical arousal is important for emotional awareness/ emotion regulation	*Psychological theory* (design team)	Expanded functionality of app: adjusting code and visualization to include levels of PCEA below the user's personal mean HR.	
Functionality *(basic)*	Changing the watch face within the smartwatch-app is unintuitive, cumbersome and a wrong action by the user results in the app stop functioning	Primary users (1)<br>Secondary users (4)	Sense-IT supports native watch faces for Wear OS	
Functionality *(basic)*	The different options in the settings menu were not clear to the user	Expert users (3)	Decision to make the settings menu password protected, only to be accessed by a supervising expert and not by the main user. This decision was made upon considering that these options require specific knowledge of the system, the algorithm used for calculating PCEA levels, and the effect of changing parameters. Also, once the system is set correctly to the personal profile of a primary user, these settings are not meant to be changed – willingly or accidently.	
Functionality *(advanced)*	Suggestion to add camera functionality to add photos of locations/situations	Expert Users (1)	Not implemented due to privacy issues regarding such an option	
Functionality *(advanced)*	Suggestion to further integrate the use of emoticons/emoji in the app	Expert Users (1)	Not implemented due to ambiguousness of meaning emoji can have	*Future work:*<br>Option to indicate experienced or subjective emotional state by select emoji
Functionality *(advanced)*	There is a need for advanced graphical visualization options to guide the user in monitoring: figures, graphs and timelines for easy use and integration in therapy	Primary users (5)<br>Secondary users (1)<br>Experts users (3)		*Future work:* development of web interface for use by primary and/or secondary users.<br>Alternate version with updated graphics and animations is under way; further advanced forms of visualization are planned to be integrated in the web-application
Functionality *(advanced)*	There is a need for an option to add personal notes to measurements as memory aid	Primary users (5)	Options to add notes to measurement, option to add miscellaneous notes	
Functionality *(advanced)*	Sharing of data between primary users and secondary users	Primary users<br>Secondary users		Option is built-in in code, not yet operational.
Functionality *(advanced)*: *Personalization*	Sensitivity by which system detects changes and notifies the user is experienced too low by some, yet correct by others: there is a need for an option to customize settings to better fit user profiles	Primary users (2)	Option to set three levels of sensitivity; option to manually set values of mean HR and standard deviation of mean HR	
Functionality *(advanced)*: *Personalization*	Users' preferences largely differ regarding the way the level of PCEA is visualized: some prefer more straightforward and clear visualization, others express a need for a more inconspicuous and unobtrusive GUI. This signals a strong need for personalization options	Primary users (4)	Inclusion of 4 different watch faces.	*Future work:* option to set colours and adjust lay-out of smartphone app. Adding of more watchfaces.<br><br>Development of multiple versions of the app with GUI's for specific use case scenarios / health settings
Functionality *(advanced)*: *Personalization*	Further options to customize/personalize the look and feel of the app such as changing the color scheme of the app, will improve user experience	Expert users (2)	Added the option to enter a personalized text, which is added to an entry in the timeline of the smartphone app when the PCEA reaches a certain level. This level is also customizable.	*Future work:* planning to add a user accessible settings menu which provides the primary users options to change settings that affect the 'look and feel' of the app
