# Supplementary material for: Development of an Ambulatory Biofeedback App to Enhance Emotional Awareness in Patients with Borderline Personality Disorder: Multicycle Usability Testing Study
Source: JMIR Mhealth Uhealth. 2019 Oct 15;7(10):e13479. doi: 10.2196/13479 (PMC6913718; doi:10.2196/13479)
Supplement: Multimedia Appendix 2 [file mhealth_v7i10e13479_app2.pdf]

Sense IT continued Questionnaire 1st iteration

Total of 26 questions.

Introduction

Scelta and the University of Twente are in the process of developing a new treatment intervention in which mobile technology is used to aid in the process of learning to better recognize emotions. To be able to achieve the best possible results, it is important to involve potential end-users in the design process. It helps in catering to the needs and requirements of future users.

As an envisioned user of such an emotion recognition intervention, you are invited to share both positive and negative points about the concept and comment on how you believe the intervention should be and look like. Be frank, there is no need to tone down any of your comments.

Use context

In this section, we would like to find out more about the setting and situations in which you use the Sense-IT

1. What is your level of experience with regard to the use of computers, smartphones, and other technology and digital applications on it like the internet and social media?

Mark only one oval.

	1	2	3	4	5	
Little experience	⬭	⬭	⬭	⬭	⬭	a lot of experience

2. **In what settings and for what purposes do you use aforementioned technology and digital applications?**

E.g. for personal use, at work, school, et cetera

....

....

....

....

....

3. **What is your estimate of the average level of experience of an average person in treatment here with regard to the use of computers, smartphones, and other technology and digital applications on it like the internet and social media?**

Mark only one oval.

	1	2	3	4	5	
Little experience	⬭	⬭	⬭	⬭	⬭	A lot of experience

4. **To what extent is the use of aforementionted technology and digital applications part of the therapy here? What do/did you learn to use, and from whom?**

....

....

....

....

....

5. **Are the things you'd expect or want from a 'mobile tech supported' emotional awareness training in any way different than in case of a non-technologically supported emotional awareness training? If so, please tell us.**

....

....

....

....

....

Experiences (general)

In this section, we would like to find out more about how the experience of using the Sense-IT app was for you.

6. **Throughout the day, where you consciencely aware of the fact that your biosignals where being monitored?**

Mark only one oval.

- ◯ Yes
- ◯ No

7. How did you experience the use of the technology

Mark only one oval.

	1	2	3	4	5	
Annoying/unpleasant	◯	◯	◯	◯	◯	Fine/ pleasant

8. Do you have other remarks regarding the use of the bio sensing technology you would like to share?

Answering this question is optional.

....

....

....

....

....

9. **What would be a primary argument (or arguments) to decide to use the technology and the app as a tool in learning to better recognize emotions? What would be a decive argument (arguments) for you to decide to not use the technology/app?**

....

....

....

....

....

10. **At what settings/locations/hours would you say the technology and app could and/or should be used, if it was up to you to decide?**

E.g. All day, at certain therapies, in the evenings, in the weekend, inside the clinic, outside of the clinic, during leaves, ...

....

....

....

....

....

Experiences (interfaces)

In this section, we would like to find out more about how you experienced the app's interfaces.

11. **How easy/difficult was it for you to work with the interface on the smartwatch?**

Tip: If familiar with the use of other mobile applications, you can compare the app's interface with the use of those

Mark only one oval.

	1	2	3	4	5	
Difficult	◯	◯	◯	◯	◯	Easy

12. **How easy/difficult was it for you to work with the interface on the smartphone?**

Tip: If familiar with the use of other mobile applications, you can compare the app's interface with the use of those

Mark only one oval.

	1	2	3	4	5	
Difficult	◯	◯	◯	◯	◯	Easy

13. **How stable and reliable did you think the interface was?**

Mark only one oval.

	1	2	3	4	5	
Not reliable	◯	◯	◯	◯	◯	Reliable

14. **Did using the interface had an effect on you (your mood, your behavior, et cetera)?**
E.g. Did it have a relaxing effect on you, or did it work on your nerves, ...?

....

....

....

....

....

15. **There are four watch faces for the smartwatch app. Which design did you like the most?**
Pictures of the different designs are provided on a separate sheet
Mark only one oval.

- ◯ Design 1
- ◯ Design 2
- ◯ Design 3
- ◯ Design 4

16. **Can you explain why you liked the one you chose the most.**

....

....

....

....

....

17. **Is there also a design that you didn't like? If so, could you explain why?**

....

....

....

....

....

18. **What design would most other patients probably like most?**
Mark only one oval.

- ◯ Design 1
- ◯ Design 2
- ◯ Design 3
- ◯ Design 4

19. **Please explain why you think this design would appeal the most to most users**

....

....

....

....

....

Experiences (notifications)

In this section, we would like to find out more about your thoughts and opinions with regard to the notifications sent by Sense-IT

20. **To what extent were the notifications sent in a inobtrusive manner?**
e.g. did you had the idea that others were able to tell when you received a notification?
Mark only one oval.

	1	2	3	4	5	
obtrusive	◯	◯	◯	◯	◯	inobtrusive

21. **In what way, did the the app affect you when it comes to the use of notifications?**
E.g. Did it have a relaxing effect on you, or did it work on your nerves, ...?

....

....

....

....

....

22. **What's your opinion/experience with regard to the number of notifications you received on registered changes in your arousal level?**
Please indicate whether the number of notifications was adequate/inadequate, helpful/annoying, et cetera.

....

Some general questions

Some final questions that can help us in improving the app.

23. **Do you see any risks in the actual use of this app/new intervention? What rare they?**

(E.g. Potential misuse by certain other people, overly reliance on the technology, misinterpretation of feedback and making wrong decisions because of it, disruption of general processes in therapy?)

....

....

....

....

....

24. **What kind of information (such as certain biological variables) do you think are important to monitor and use in Sense-IT, the emotional awareness training app?**

E.g. self reports (through questionnaires or otherwise), subjective indices, skin conductance measures, heart rate, ... ? As much as possibly can be measured (for a detailed view and/or most accurate feedback), as little as necessary to give some estimate of my emotional arousal, ... ?

25. **Regarding the privacy of your data, what is important regarding who get's access and who not, and to what data?**

To what extent should data collected with the app be kept completely private? So, to what extend it's okay by you to share data with your main therapist, other therapists involved in your treatment, other patients in the program/your group, technical support, researchers, others?

26. **How many months have you been in therapy here (0-9)**

Powered by

Google Forms
