# Supplementary material for: Development of an Ambulatory Biofeedback App to Enhance Emotional Awareness in Patients with Borderline Personality Disorder: Multicycle Usability Testing Study
Source: JMIR Mhealth Uhealth. 2019 Oct 15;7(10):e13479. doi: 10.2196/13479 (PMC6913718; doi:10.2196/13479)
Supplement: Multimedia Appendix 3 [file mhealth_v7i10e13479_app3.pdf]

Sense IT Continued Questionnaire 2nd iteration

Total of 16 questions.

Introduction

Scelta and the University of Twente are in the process of developing a new treatment intervention in which mobile technology is used to aid in the process of learning to better recognize emotions. To be able to achieve the best possible results, it is important to involve potential end-users in the design process. It helps in catering to the needs and requirements of future users.

As an envisioned user of such an emotion recognition intervention, you are invited to share both positive and negative points about the concept and comment on how you believe the intervention should be and look like. Be frank, there is no need to tone down any of your comments.

1. What where your experiences on using the application today?

We would love to hear all your comments and remarks on your experiences and other things that you think that's important.

....

....

....

....

....

....

....

....

....

....

Experiences (interfaces)

In this section, we would like to find out more about your thoughts and opinions about the watch face designs of on the smartwatch.

2. **How easy/difficult was it for you to work with the interface on the smartwatch?**

Tip: If familiar with the use of other mobile applications, you can compare the app's interface with the use of those

Mark only one oval.

	1	2	3	4	5	
Difficult	◯	◯	◯	◯	◯	Easy

3. **How easy/difficult was it for you to work with the interface on the smartphone?**
Tip: If familiar with the use of other mobile applications, you can compare the app's interface with those
Mark only one oval.

	1	2	3	4	5	
Difficult	◯	◯	◯	◯	◯	Easy

4. **How stable and reliable did you think the interface was?**
Here you can indicate the extent to which you think the app is reliable and stable
Mark only one oval.

	1	2	3	4	5	
Not reliable	◯	◯	◯	◯	◯	Reliable

5. **There are four watch faces for the smartwatch app. Which design did you like the most?**
Pictures of the different designs are provided on a separate sheet
Mark only one oval.

◯ Design 1
◯ Design 2
◯ Design 3
◯ Design 4

6. **Please explain why you liked the one you chose the most.**

....
....
....
....
....

7. **Is there also a design that you didn't like? If so, could you explain why?**

....
....
....
....
....

Experiences (notes)

In this section, we would like to find out more about your thoughts and opinions about the fucnction to add notes.

8. **Did you use the function of adding notes to the recorded changes in the smartphone app?**

Mark only one oval.

- ( ) Yes
- ( ) No

9. **To what extent do you believe it is useful to add self report notes in the app for personal use?**

Mark only one oval.

	1	2	3	4	5	
Not useful	( )	( )	( )	( )	( )	Useful

10. **To what extent do you believe it is useful to add self report notes in the app for use in therapy sessions?**

e.g. to what extent do you think it's useful in a session with your therapist, or in group therapy?

Mark only one oval.

	1	2	3	4	5	
Not useful	( )	( )	( )	( )	( )	Useful

11. **Can you explain the answer you give to the previous question?**

....

....

....

....

....

12. **In receiving and/or adding feedback from/to the app, what do like (most): working with plain text or with emoticons?**

....

....

....

....

....

13. **What types of personal contact with your therapist do you think you'll need to effectively work with the app and get out the most of it? (e.g. in person, via chat, email, calling, ...)**

....

....

....

....

....

14. **Regarding the privacy of your data, what is important regarding who get's access and who not, and to what data?**

To what extent should data collected with the app be kept completely private? So, to what extend it's okay by you to share data with your main therapist, other therapists involved in your treatment, other patients in the program/your group, technical support, researchers, others?

....

....

....

....

....

15. **What information should be automatically and actively shared with your therapist by the app (if any)?**

....

....

....

....

....

One final question

16. **How many months have you been in therapy here (0-9)**

Knowing this can help us in correctly understanding and processing the feedback.

....
